# Exploring the equity of GP practice prescribing rates for selected coronary heart disease drugs: a multiple regression analysis with proxies of healthcare need

**DOI:** 10.1186/1475-9276-4-3

**Published:** 2005-02-08

**Authors:** Paul R Ward, Peter R Noyce, Antony S St Leger

**Affiliations:** 1 Section of Public Health, School of Health and Related Research (ScHARR), University of Sheffield, Sheffield, UK; 2 School of Pharmacy and Pharmaceutical Sciences, University of Manchester, Manchester, UK; 3 Evidence for Population Health Unit, School of Epidemiology and Health Sciences, University of Manchester, Manchester, UK

## Abstract

**Background:**

There is a small, but growing body of literature highlighting inequities in GP practice prescribing rates for many drug therapies. The aim of this paper is to further explore the equity of prescribing for five major CHD drug groups and to explain the amount of variation in GP practice prescribing rates that can be explained by a range of healthcare needs indicators (HCNIs).

**Methods:**

The study involved a cross-sectional secondary analysis in four primary care trusts (PCTs 1–4) in the North West of England, including 132 GP practices. Prescribing rates (average daily quantities per registered patient aged over 35 years) and HCNIs were developed for all GP practices. Analysis was undertaken using multiple linear regression.

**Results:**

Between 22–25% of the variation in prescribing rates for statins, beta-blockers and bendrofluazide was explained in the multiple regression models. Slightly more variation was explained for ACE inhibitors (31.6%) and considerably more for aspirin (51.2%). Prescribing rates were positively associated with CHD hospital diagnoses and procedures for all drug groups other than ACE inhibitors. The proportion of patients aged 55–74 years was positively related to all prescribing rates other than aspirin, where they were positively related to the proportion of patients aged >75 years. However, prescribing rates for statins and ACE inhibitors were negatively associated with the proportion of patients aged >75 years in addition to the proportion of patients from minority ethnic groups. Prescribing rates for aspirin, bendrofluazide and all CHD drugs combined were negatively associated with deprivation.

**Conclusion:**

Although around 25–50% of the variation in prescribing rates was explained by HCNIs, this varied markedly between PCTs and drug groups. Prescribing rates were generally characterised by both positive and negative associations with HCNIs, suggesting possible inequities in prescribing rates on the basis of ethnicity, deprivation and the proportion of patients aged over 75 years (for statins and ACE inhibitors, but not for aspirin).

## Background

The aim of this paper is to provide data on the equity of general medical practitioner (GP) prescribing rates for coronary heart disease (CHD) drugs. Since CHD represents a major cause of premature mortality in the Western world, it is vital that those populations with the highest need for CHD drugs actually receive them. Whilst there is a large literature on inequities in the provision of a number of other health care services and treatments, the equity of GP practice prescribing has received little attention. Therefore, this study was an attempt initiate the development of an evidence-base and to provide data on the equity of GP practice prescribing rates.

### Conceptualisation and definition of the equity of prescribing

There are large literatures around how to define, operationalise and measure equity in relation to health care services [1-3], although equity is generally taken to mean 'fair' or 'just'. Equity has been divided into three domains: equal *access *to health care for people in equal need; equal *treatment *for people in equal need; and equal *outcomes *for people in equal need [1]. Whilst this is a simplification of the nature of equity, it is useful in delineating the various domains in which inequities may arise.

The current paper is focussed around the equal prescribing (i.e. equal *treatment*) for people in equal need. Using the example of the current study, an analysis of equity would assess the differences in prescribing rates provided to the population of one GP practice compared to another GP practice, weighted to take account of the levels of need for CHD drugs in their patient populations. Therefore, it would be equitable to have higher prescribing rates for populations with higher levels of health care need and lower prescribing rates for populations with lower levels of health care need. However, it would be inequitable to have higher prescribing rates for populations with lower levels of health care need and lower prescribing rates for populations with higher levels of health care need. The identification of populations where prescribing was deemed inequitable could then be targeted for further resources aimed at redressing the balance between prescribing rates and health care need.

### Previous research on the equity of GP practice prescribing

A recent paper by the authors questioned the equity of GP practice prescribing rates for a range of CHD drugs[4] and highlighted the contemporary relevance of the 'inverse care law'[5] in the context of GP prescribing. That paper presented the findings of bivariate correlations between prescribing rates and healthcare needs indicators (HCNIs). One of the inherent problems with bivariate analysis is that prescribing rates are likely to be associated with a number of HCNIs. Therefore, the purpose of this paper is to present the findings of multivariate regression analyses between prescribing rates and the HCNIs and ultimately to examine the independent associations between prescribing rates and HCNIs. In doing so, this paper sets a benchmark for future studies aimed at assessing the effectiveness of the National Service Framework for CHD in developing CHD services commensurate with healthcare need [6].

There is a growing body of research which has highlighted large variations in overall prescribing rates between GP practices, which are only partially explained by factors other than health care need [4,7-11]. Statin prescribing has been shown to vary between health authorities and GPs [12-15] and between patients on the basis of gender [13,16-18], demographics [13,19], ethnicity [20] and deprivation [21]. Prescribing rates of beta-blockers have also been found to be lower in patients aged over 75 years and in minority ethnic groups [22,23].

Studies attempting to 'explain' the variation in statin prescribing rates have been modest, with most studies explaining around 20 per cent of the variation [13,15,21,24]. In addition, one study explained around 40 per cent of the variation in prescribing rates for all cardiovascular drugs [21]. The prevalence of CHD explained 12 per cent of the variation in statin prescribing in men, and 7 per cent in women [13], deprivation explained 14 per cent [15], and a combination of nitrate prescribing rates and population aged between 35 and 74 years explained 18 per cent [24]. In addition, the indicative prevalence of CHD was moderately correlated with prescribing rates for statins, and was the most important variable in the multiple regression model [21]. However, characteristics of GP practices such as their training status, the number of GPs, or their single-handed status (i.e. whether or not they are 'lone' GPs or work in a multi-partner practice) have been found to have no relationship with prescribing rates for statins [15,24]. Therefore, the majority of variations in statin prescribing rates in addition to prescribing rates for other CHD drugs remain unexplained.

### Context and setting for the study

The planning and provision of health care to local populations in England is now the role of primary care trusts (PCTs). Essentially, PCTs are organisations whose main responsibilities are around developing, commissioning and providing services, which are targeted to the needs of local people, and ultimately to improve the health (and reduce health inequalities) of local people [25,26]. PCTs have taken over these responsibilities from health authorities, which no longer exist [27] and are responsible for spending 75 per cent of the overall NHS budget in England [28].

This study was undertaken in 4 PCTs in England (referred to as PCT1, PCT2, PCT3, and PCT4 throughout this paper), which included 132 GP practices (PCT1 had 50 GP practices, PCT2 had 24, PCT3 had 31 and PCT4 had 27). In terms of patient populations, we excluded patients aged under 35 years, since prevalence of CHD is particularly low in this age group. In total, there were 353,897 registered patients aged over 35 year across all 4 PCTs.

## Methods

In order to analyse the equity of prescribing for CHD drugs, we firstly needed to gather data on, and then develop rates for both GP practice prescribing and health care need (called health care needs indicators (HCNIs) in this paper). The data sources and methods used to develop prescribing rates and HCNIs have been outlined in previous papers by the authors [4,29,30], although a brief précis will be presented here. Local Research Ethics Committee approval was sought and granted for this study.

### Developing prescribing rates

When an NHS prescription is dispensed in primary care, the prescription form (FP10) is sent to the Prescription Pricing Authority (PPA) for processing. The PPA collates these data and provides them to GP practices and PCTs in the form of Prescribing Analysis and Cost (PACT) data. PACT data are available for all GP practices in England, and allow detailed interrogation in terms of drugs prescribed along with their dosages, pack sizes and formulations. For example, for a specific time period, we can collect data on which statins were prescribed by a GP practice in addition to the dosages and pack sizes. This allows for a complex and timely analysis of PACT data. Useful critiques of PACT data can be found elsewhere [31,32].

PACT data were obtained for all GP practices in the 4 PCTs for the 12-month period October 1999 to September 2000. These data were collected for statins, ACE-inhibitors, beta-blockers, aspirin, and bendrofluazide (a full list of drugs obtained are listed in Appendix A). These drug groups were chosen because they represent major drug groups recommended for the prevention (primary and secondary) of coronary heart disease (CHD) in the United Kingdom (UK) [6]. Using prescribing rates for aspirin was potentially difficult, since it can also be purchased over the counter (OTC) in community pharmacies and therefore may not represent the totality of aspirin use within populations [33,34]. It has also been found that non-prescription aspirin use was higher in men aged under 65 years, and also in more affluent areas [35]. Therefore, PACT data may underestimate actual aspirin use within the community. Nevertheless, given the importance of aspirin within the management and prevention of CHD [17,36,37], it was postulated that prescribing rates for aspirin may reflect CHD prevalence within GP practice populations, at least as well or even better than prescribing rates for the other CHD drugs.

The numerator in all prescribing rates was based on a measure of prescription volume, as opposed to prescription cost. The validity of using the number of prescription items or total cost as a measure of prescribing volume has been called into question [38,39] since it does not specify the quantity of prescription medication (e.g. number and/or dosage of tablets). Therefore, a measure of prescription volume which calculates the total number of grams prescribed is much more useful. The main options available are defined daily doses (DDDs) [40,41] and Average Daily Quantities (ADQs) [40,42,43]. The Prescribing Support Unit website provides up to date lists of DDDs  and ADQs  for all drugs for which they have been developed. Within this study, total ADQs were used as the unit of analysis since they represent prescribing practices in the UK, as opposed to DDDs which represent prescribing practices internationally.

The denominator was the total registered (and resident) patient population aged over 35 years. This age group was chosen since the prevalence of CHD is particularly low in people aged less than 35 years [44].

### Developing health care needs indicators (HCNIs)

In total, 24 HCNIs were developed for each GP practice in this study, and all of these were entered into multiple regression models (all HCNIs are outlined in Appendix B). These HCNIs reflect patient demographics, ethnicity, socio-economic status, long term limiting illness, CHD mortality and CHD morbidity. However, the only HCNIs discussed here are those included in the final regression models (see Table 1).

**Table 1:** Descriptions of statistically significant variables in final regression models

**Descriptor used in text and Table 4**	**Description**
CHD HES rate	6-year crude rate of CHD hospital procedures per 1000 patients (i.e. proxy for CHD morbidity). Data source was the General Practice Research Database.
% patients aged 55–74	Proportion of patients in GP practice population aged 55–74 years. Data source was the individual GP practices.
% patients aged >75	Proportion of patients in GP practice population aged over 75 years. Data source was the individual GP practices.
LISI score	Low Income Scheme Index (LISI) score which is the proportion of prescriptions in GP practices which were exempt from payment due to low income (i.e. proxy for deprivation). Data source was the individual GP practices.
Ethnicity	Proportion of patients in GP practice population defined as South Asian. Data source was the 1991 Census.

In order to assess the equity of prescribing for CHD drugs, we needed to develop a set of variables which measured (or acted as proxies for) the health care need in GP practice populations. In terms of coronary heart disease (CHD), there are a number of identifiable risk-factors, all of which increase the risk of CHD morbidity and/or mortality. However, readily available data are not available for a number of these risk-factors, and therefore could not be explored within this study. Nevertheless, data were available on the following risk-factors: age, gender ethnicity, socio-economic status. Evidence on the importance of these risk-factors for assessing health care need is presented below.

Rates of CHD related morbidity and mortality increase dramatically with age [45,46]. For example, the prevalence of angina in England was 1 per cent in people aged 16–44 years, 3 per cent in those aged 45–54 years, although this increased to 10 per cent in those aged 55–64 years, 16 per cent in those aged 65–74 years and 18 per cent in those aged over 75 years [47].

Rates of CHD mortality and morbidity in the UK are generally seen to be higher for people from South-Asian groups, than from white-English people, and much lower for people born in the Caribbean. It has been suggested that the mortality rate for South Asians is 50 per cent higher than for the general population [48]. In the UK, the standardised mortality rates for CHD in South-Asian men was 146, compared to 100 for all men and just 46 for men born in the Caribbean [49]. It is also recognised that there are differences within South Asian groups, especially between Indians, who generally experience better health, and Pakistanis or Bangladeshis, who have generally worse health [50-53]. However, reliable data on CHD morbidity or mortality down to these more complex ethnic groupings are not readily available, and any estimates are, at best, imprecise [50]. Nevertheless, data on the ethnic minority profiles of GP practice populations may prove very useful in determining the need for CHD drugs.

There is an extensive literature on socio-economic inequalities in CHD mortality and morbidity [54-57]. Whilst rates of CHD have been declining in the UK for almost 20 years, they have not been falling as fast as countries such as Australia and the United States [46]. Declining CHD mortality rates are only partially explained by reductions in established cardiovascular risk factors [58-60] and it is possible that general social and economic improvement over time has contributed to this trend [61]. However, it is noteworthy that these benefits have not been observed by all of the socio-economic groups within the UK [62]. For example, deaths from CHD in men in the highest social class have halved in the past 20 years, but remain almost unchanged among men in the lowest social class [60]. Between 1986 and 1992, people from the highest social classes had a rate of 160 CHD deaths per 100,000 population, whereas the rate for the lowest social classes was 266 per 100,000 [49]. In addition, a 22-year follow-up study on the relationship between socioeconomic status and CHD in middle-aged men [63] found that irrespective of length of follow-up, lower social classes had a clearly increased risk of fatal CHD – after 8 years they had a 69 per cent increased risk, which dropped to 67 per cent after 15 years and 59 per cent after 22 years. Therefore, socio-economic status is significantly related to CHD mortality and morbidity, and as such, represents an important indicator of health care need for CHD drugs.

Overall, the age, ethnicity and socio-economic status are all important factors in shaping the epidemiology of CHD in the UK, and as such, are important variables for use in developing the HCNIs in this study. The actual development of HCNIs relating to these risk factors are outlined below, in addition to additional HCNIs related more directly to CHD morbidity and mortality.

Some HCNIs were collected directly from GP practice lists (proportion of patients aged over 75 years and proportion of patients aged 55–74 years), some have been calculated for GP practices (Low Income Scheme Index (LISI) score) [64], and others were calculated from data directly attributable to GP practices (data based on hospital episode statistics). However, due to the lack of information available from GP practices on variables such as socio-economic status and ethnicity (in this study, this refers to South Asian groups) of patients, a number of HCNIs were developed which estimated this from data such as the 1991 Census and Local Authority statistics. The method of patient weighted attribution was used to develop these estimates using data at enumeration district level (small geographical areas), whereby the postcodes of patients were linked to enumeration districts. Data on the enumeration districts of all registered patients were then aggregated for each GP practice and divided by the total registered population in order to provide a patient-weighted score. In this way, data from the Census were directly applied to GP practice populations. Further information and critiques of this method can be found elsewhere [65-67]. Whilst 1991 Census data may be regarded as rather old now (and has since been superseded by 2001 Census data), these were the only data available at the time of the study, since GP practices do not routinely collect data on the ethnicity of patients. Nevertheless, the HCNI relating to ethnicity may be seen with caution in this paper.

Demographic HCNIs were developed directly from GP practice list data, and these relate to the proportion of patients aged 55–74 years, and the proportion aged over 75 years. Both of these demographic groups are indicators of health care need for CHD drugs [44]. The Low Income Scheme Index (LISI) was used as a proxy for low income since it represents the proportion of prescriptions which are exempt from prescription charges due to low income [64]. The proportion of patients from South Asian groups was estimated for each GP practice using data from the 1991 census.

Data were also obtained from hospital episode statistics (HES) on specific hospital procedures (coronary artery bypass graft (CABG), percutaneous transluminal angioplasty (PTCA), and coronary angiogram) and diagnoses (primary diagnosis of CHD at discharge). Although HES relate to the supply, as opposed to need for health care services [68], it was hypothesised that in the absence of other CHD morbidity data, they may represent a useful proxy of CHD morbidity in GP practice populations. Due to the low numbers of procedures and diagnoses within a GP practice population, data were aggregated for 6 years (1995 to 2000 inclusive). Crude rates (per 1000 patients aged over 35 years) were calculated for CHD HES, which represents the CHD procedures + CHD diagnoses.

As outlined in Appendix B, a number of other variables relating to CHD mortality and morbidity were entered into the regression models, although these were not statistically significant. Indeed, as outlined in a previous paper by the authors [4], bivariate analyses revealed that associations between prescribing rates and standardised mortality rates for CHD were often not statistically significant and in some cases had a negative association. Therefore, prescribing rates and CHD mortality rates do not exhibit a strong relationship, adding to the suggestion of an inequity in prescribing rates.

### Data analysis

Multiple linear regression modelling was undertaken for each drug group in each PCT in addition to the combined dataset. The dependent variable in each model was the respective prescribing rate and the independent variables were all 24 HCNIs developed in the study. The forward-stepwise approach was taken but not slavishly adhered to since considerations of model coherence and plausibility were taken into account when seeking the most parsimonious models. The final regression models only contained the HCNIs which had statistically significant (p < 0.05) independent associations with the dependent variable, and each model was checked for collinearity and normality of residuals. Overall, for each multiple regression model, all 24 HCNIs were entered as independent variables, and the final model included only those variables that were statistically significant and added to the fit of the model.

## Results

Descriptive findings will be presented first, in order to contextualise the findings from the multiple regression analyses.

### Variations in prescribing rates and health care needs indicators (HCNIs)

Table 2 provides details of variations between primary care trusts (PCTs) in prescribing rates for each of the study drug groups. PCT1 generally had the highest median prescribing rates, followed by PCT2, with the lowest prescribing rates occurring in PCT3 and PCT4. The median ADQs per patient over 35 years for all study CHD drugs combined were 105 in PCT1, 93 in PCT2, 90 in PCT3, and 84 in PCT4.

**Table 2:** Variation in prescribing rates by primary care trust (PCT)

**Drug group**		**PCT1**	**PCT2**	**PCT3**	**PCT4**
**Statins**	**Min**	11.05	9.56	6.86	6.44
	**Max**	47.23	32.12	46.60	45.76
	**Median**	22.88	19.65	18.08	17.36
	**IQR**	8.20	11.20	8.40	7.24

**Aspirin**	**Min**	14.62	15.34	4.94	9.6
	**Max**	44.97	46.40	46.63	45.65
	**Median**	29.27	27.35	25.33	30.87
	**IQR**	7.40	16.5	7.60	18.85

**Beta-blockers**	**Min**	5.04	6.38	3.94	3.43
	**Max**	23.92	29.04	23.91	26.19
	**Median**	12.84	12.38	12.32	12.64
	**IQR**	4.61	7.70	7.41	10.11

**ACE inhibitors**	**Min**	15.47	9.85	4.20	4.69
	**Max**	48.40	39.25	32.97	35.69
	**Median**	26.43	19.76	16.59	19.19
	**IQR**	8.82	11.17	9.37	11.29

**Bendrofluazide**	**Min**	4.19	1.63	1.63	0.60
	**Max**	35.61	29.55	32.90	22.91
	**Median**	14.42	10.85	11.04	9.01
	**IQR**	6.98	8.15	12.70	7.28

**All study CHD drugs**	**Min**	71.70	57.28	25.89	24.76
	**Max**	156.82	154.21	136.64	150.68
	**Median**	105.37	92.78	90.33	83.65
	**IQR**	28.25	57.07	32.08	51.18

PCT1 had the highest median prescribing rates for all drugs except aspirin, where PCT4 had the highest median rate. PCT3 had the lowest median prescribing rates for aspirin, beta-blockers and ACE inhibitors, and PCT4 had the lowest median prescribing rates for statins, bendrofluazide, and all study CHD drugs combined. The difference between the median prescribing rates for PCT1 and PCT4 for bendrofluazide, statins and all CHD drugs was around 4, 5, and 21 ADQs per patient over 35 years. Therefore, an average GP practice in PCT1 with 3000 registered patients over 35 years, prescribed 12000 more ADQs for bendrofluazide, 15000 more ADQs for statins, and 63000 more ADQs for all study CHD drugs. In addition, a similar GP practice in PCT1 also prescribed almost 30000 more ADQs for ACE inhibitors than a comparably sized GP practice in PCT3.

An initial overview of the health care needs of the PCTs suggests that PCT4 had the highest levels of CHD related health care needs within the study, whereas PCT1 had the lowest health care needs. PCT4 was the most deprived of all PCTs, had the highest proportions of patients aged over 75 years, and the highest median score for standardised mortality rates (SMR) for CHD. In contrast, PCT1 may be seen as the 'least needy' of all PCTs on the basis of the HCNIs developed in this study. PCT1 was the least deprived, had the lowest proportions of South Asian groups, had the lowest median SMR and the lowest median rate of CHD hospital procedures.

These overall descriptive findings are indicative of inequitable prescribing rates, whereby PCT1 had high prescribing rates and low comparative health care needs, whereas PCT4 had low prescribing rates and high comparative health care needs. However, these are purely descriptive and do not take into account the multiple risk factors for CHD or the interactions between HCNIs. Therefore, findings from the multiple regression analyses are presented below.

### Multiple regression analysis

Table 3 presents the percentage of variation explained in each of the models (i.e. R^2 ^* 100), Table 1 presents a description of the HCNIs included in the final regression models and Table 4 presents details for the combined dataset in terms of the HCNIs included in each model, their standardised beta coefficients and their contribution (in terms of percentage variation explained) to the total R^2 ^of the model.

**Table 3:** Percentage of variation in prescribing rates explained by HCNIs

	**PCT1**	**PCT2**	**PCT3**	**PCT4**	**Combined**
**Statins**	24.5	58.3	31.3	50.5	24.9
**Aspirin**	44.2	88.2	62.3	51.7	51.2
**ACE inhibitors**	No Model	55.3	26.6	45.8	31.6
**Beta-blockers**	28.3	42.4	46.1	41.1	27.1
**Bendrofluazide**	15.6	53.9	40	54.5	24.1

**Table 4:** Regression models for combined dataset

**Drug group**	**R^2^**	**Needs indicator**	**Beta coefficient**	**% explained**
**Statins**	.249	CHD HES rate	.350	14.7
		% patients aged >75	-.240	4.2
		Ethnicity	-.233	3.1
		% patients aged 55–74	.199	2.9

**Aspirin**	.512	CHD HES rate	.579	32.9
		LISI score	-.261	10.4
		% patients aged >75	.347	8.2

**ACE inhibitors**	.316	Ethnicity	-.395	25.3
		% patients aged >75	-.170	3.4
		% patients aged 55–74	.248	2.9

**Beta-blockers**	.271	CHD HES rate	.411	19.7
		% patients aged 55–74	.274	7.4

**Bendrofluazide**	.241	LISI score	-.261	13.1
		CHD HES rate	.171	6.6
		% patients aged 55–74	.274	4.4

**All CHD drugs**	.454	LISI score	-.303	22.7
		CHD HES rate	.461	20.0
		% patients aged 55–74	.242	2.7

Regression models generally explained much more variation in prescribing rates in PCT2 than any other PCT. For example, in PCT2, modelling explained around 55 per cent of the variation in prescribing rates for ACE inhibitors and bendrofluazide, almost 60 per cent for statins and almost 90 per cent of variation in aspirin prescribing rates. Multiple regression models explained around 25 to 55 per cent of the variation in prescribing rates in the combined dataset, around 25 to 60 per cent in PCT3, and around 40 to 55 per cent in PCT4. PCT1 had the lowest R^2 ^values in general, and no model was derived for ACE inhibitors. Given the reduced explanatory power of the regression models in PCT1, when regression modelling was undertaken for the combined dataset, the R^2 ^values were much lower than those for PCT2, and generally lower than those for both PCT3 and PCT4.

In general, regression modelling explained more variation in prescribing rates for aspirin than for the other drug groups. Not including PCT1, regression modelling explained between 50 and 90 per cent of the variation in aspirin prescribing rates. Comparative percentages were between 30 and 60 per cent for statins, 30 and 45 per cent for beta-blockers, and 25 and 55 per cent for ACE inhibitors and bendrofluazide.

Table 4 presents details of the regression models derived for each drug group in the combined dataset. Many of the models in Table 4 are quite similar in terms of the HCNIs included within them, with all models (except for ACE inhibitors) including the rate of CHD HES (positive association) and all models (except for aspirin) including the proportion of patients aged between 55 and 74 years (positive association). In addition, the LISI score was included in the models for aspirin, bendrofluazide and all CHD drugs (negative association).

The models for statins, aspirin and ACE inhibitors also included the proportion of patients aged over 75 years although this indicator had a negative association with prescribing rates for statins and ACE inhibitors, and a positive association with prescribing rates for aspirin. This suggests that GP practices with higher proportions of patients aged over 75 years have lower prescribing rates for statins and ACE inhibitors, and higher prescribing rates for aspirin.

The CHD HES rate varied considerably between models, in terms of the amount of variation explained. For example, it explained 33 per cent of the variation in aspirin prescribing rates, 20 per cent of the variation in prescribing rates for all CHD drugs and beta-blockers, 15 per cent of the variation in statin prescribing rates, and 7 per cent of the variation in prescribing rates for bendrofluazide.

The LISI score explained 23 per cent of the variation in prescribing rates for all CHD drugs, 13 for bendrofluazide and 10 per cent for aspirin. In addition, the proportion of patients aged between 55 and 74 years explained between 2.5 and 7 per cent of the variation for statins, ACE inhibitors, beta-blockers, bendrofluazide, and all CHD drugs.

## Discussion

The rate of CHD hospital procedures (CHD HES) explained some of the variation in prescribing rates and therefore, prescribing rates in these instances were positively related to health care need (i.e. equitable). These findings concur with other studies whereby positive relationships were found between rates of PTCAs and both aspirin prescribing [36] and statin prescribing [36,69,70]. These may suggest that prescribing rates are positively related to healthcare need, although as already outlined, HES may actually reflect supply or demand, rather than need per se. Nevertheless, within this study, HES were the best data available as proxies for CHD in the GP practices, and therefore, we suggest that these prescribing rates reflect CHD prevalence, and hence healthcare need. GP practices in the UK are now developing CHD registers, which may be used in the future as proxies of CHD prevalence, although again, these will only reflect treated CHD, as opposed to the true prevalence of CHD in the community.

The proportion of patients aged 55–74 years was generally positively related to prescribing rates, suggesting that prescribing rates are related to health care need, since people in this age group have a higher prevalence of CHD [49]. A number of studies have found that statin prescribing is higher in this age group, than in older age groups [13,16,17,70], with one study finding that the proportion of patients aged 35–74 years explained just 5 per cent of the variation in statin prescribing rates between GP practices [24].

Whilst the previous finding is not unexpected given the higher CHD prevalence in this age group, the negative relationship between the proportion of patients aged over 75 years and some prescribing rates should be of great interest. The proportion of patients aged over 75 years had a negative relationship with statins and ACE inhibitors and a positive association with aspirin. A number of studies found that older patients were much less likely than younger patients to undergo a CABG or a PTCA[71,72] or to receive a prescription for a statin [13,16,17,70], which may result from the lack of research evidence on the efficacy of statins in elderly populations or judgements about likelihood of benefit based on age. Therefore, the negative associations between the proportion of patients aged over 75 years and rates of ACE inhibitor and statin prescribing are in line with other seemingly inequitable health care. Although the proportion of patients aged over 75 years is a proxy for CHD prevalence, it may not represent a useful proxy of the potential to benefit from statins. However, it may be suggested that prescribing rates for aspirin are higher in this age group since they are more cost-effective than statins [73]. Therefore, these drugs may be more readily prescribed to patients for whom the benefits (both clinical and cost-effectiveness) of statins have not been studied.

## Conclusion

Overall, this study has found that prescribing rates may be explained (to differing degrees between primary care trusts (PCTs) and between drug groups) by a mixture of health care need indicators (HCNIs). Whilst prescribing rates were generally positively related to the rates of hospital procedures and diagnoses, they were also negatively associated with proxies of deprivation and ethnicity and with the proportion of patients aged over 75 years. However, the relatively low R^2 ^values reveal a large amount of unexplained variation in prescribing rates. Indeed, even with the models for aspirin and all CHD drugs, there is still around 50 per cent of unexplained variation in prescribing rates.

This study cannot be used to infer inequitable prescribing by GPs, since the lower prescribing rates in areas with high proportions of South Asian and deprived groups may be due to lower utilisation of primary health care services due to social, economic or cultural barriers [74,75]. Therefore, further work needs to be undertaken in areas of deprivation and with high proportions of minority ethnic groups in order to understand the reasons for the low prescribing rates and ultimately to make CHD prescribing commensurate with healthcare need. In addition, the HCNIs used in this study are proxies for healthcare need, and may not reflect actual healthcare need. Therefore, future studies may attempt to verify our findings by using either morbidity or mortality data gathered from GP practices.

Whilst the clinical and epidemiological data on these CHD drugs has allowed for the development of evidence-based guidelines and evidence-based prescribing, this paper suggests that in practice, actual prescribing rates may not be related to health care need. Further research needs to concentrate on verifying or falsifying these claims on a more micro-level analysis (eg clinical audit in specific GP practices identified in the study) and on exploring the reasons why such a relationship exists (e.g. qualitative studies with GPs, practice nurses and patients in the identified GP practices). In addition, more work is required to understand the differences between PCTs in terms of the explanatory power of the regression models (i.e. much more of the variation in prescribing rates were explained in PCT2 than any other PCT). Such a strategy may enable educational tools to be developed which would facilitate more evidence-based prescribing, but may also identify particular patient groups who do not present symptoms of CHD (ie unmet need) and therefore may require educational outreach or targeted screening in order to increase their consultations and ultimately prescribing to these groups. Although we have focussed on drugs used in the prevention of CHD, a similar approach may be taken across different therapeutic groups of drugs, in different health care settings and in different countries in order to generate more rounded, grounded and extensive evidence on the equity of prescribing in general.

## Appendix A – List of drugs used in this study

• Aspirin (75 mg)

• Bendrofluazide^1 ^(2.5 mg)

• Statins (Atorvastatin, Cerivastatin, Fluvastatin, Pravastatin, Simvastatin)

• ACE inhibitors^2 ^(Captopril, Enalapril, Lisinopril, Ramipril, Trandolapril)

• Beta-blockers^3 ^(Atenolol, Co-tenidone^4^)

^1 ^In some countries, this may also be known as (among other names) Neo-NaClex, Bendroflumethiazide, Aprinox, Berkozide, Naturetin, Pluryl, Polidiuril, Salural, Urizde.

^2 ^The 5 ACE inhibitors represent the majority of prescribing for all ACE inhibitors

^3 ^Atenolol represents the majority of prescribing of all beta-blockers

^4 ^Co-tenidone is a combination product containing both a beta-blocker (atenolol) and a diuretic (chlorthalidone).

## Appendix B – List of health care needs indicators (HCNIs) developed during the study

• Proportion of patients aged between 55 and 74 years

• Proportion of patents aged over 75 years

• Proportion households with no car

• Proportion males who are economically inactive

• Townsend Score

• Proportion of households receiving council tax benefits

• Proportion unemployment

• Index of Multiple Deprivation

• Income Deprivation Index

• Low Income Scheme Index (LISI) score

• Standardise mortality rate (SMR) for CHD under 75 years

• 6-year crude rate of coronary artery bypass grafts (CABGs) per 1000 patients

• 6-year crude rate of percutanious transluminal angioplasty (PTCAs) per 1000 patients

• 6-year crude rate of coronary angiograms per 1000 patients

• 6-year crude rate of CHD hospital procedures (CABGs + PTCAs + angiograms) per 1000 patients

• 6-year crude rate of CHD hospital diagnoses per 1000 patients

• 6-year crude rate of CHD prevalence (diagnoses + procedures) per 1000 patients

• Regionally specific prevalence, age and sex standardised prescribing units per patient over 35 years (PASS-PUs)

• Proportion of population defining themselves as 'non-white'

• Proportion of population defining themselves as 'South Asian'

• Proportion of population over 30 with a limiting long-term illness (LLI)

• Health Deprivation Index

• Proportion of households with more than 2 cars

• Access Deprivation Index

## Competing interests

The author(s) declare that they have no competing interests.

## Authors' contributions

PW was awarded funding for this study, was involved in the conception, design and managed the day to day running of the study, undertook all data collection and analysis, and wrote the paper. PN and ASL were involved in the conception and design of the study, made active contributions in project meetings, were involved in an advisory capacity in all aspects of the project and advised on drafts of the paper. PW is the guarantor.
